# Supplementary material for: Astrocyte Senescence Impairs Synaptogenesis due to Thrombospondin‐1 Loss
Source: Aging Cell. 2026 Jan 18;25(2):e70382. doi: 10.1111/acel.70382 (PMC12813271; doi:10.1111/acel.70382)
Supplement: Supplementary file 1 — Figure S1: SAMP8 hippocampi are enriched in SA‐β‐gal+ astrocytes. (A) Quantification of the percentage of astrocytes (GFAP+/S100β+) with two or more SA‐β‐gal puncta in the stratum radiatum of SAMR1 and SAMP8 mice at 10 months. (B) Representative SA‐β‐gal positive (left pannel) and negative (right pannel) astrocytes from stratum radiatum with GFAP (red) and S100β (green) biomarkers. The slices have 40 μm of thickness. (C) Percentage of SA‐β‐gal positive astrocytes (GLAST+/ATP1B2+) in ACSA‐2 primary cultures of 2 months‐old SAMR1 and SAMP8 mice. (D) Immunostaining of SA‐β‐gal (blue), GLAST (red) and ATP1B2 (green), in hippocampal astrocytes (ACSA‐2+) of 2 months‐old SAMR1 and SAMP8 mice. (E) Percentage of SA‐β‐gal positive astrocytes (GLAST+/ATP1B2+) in ACSA‐2 primary cultures of 10 months‐old mice. (F) Immunostaining of SA‐β‐gal (blue), GLAST (red) and ATP1B2 (green), in hippocampal astrocytes (ACSA‐2+) of 10 months‐old SAMR1 and SAMP8 mice. Three independent animals and primary cultures of each strain and age were analyzed (n = 3). Data are presented as mean ± SEM. Unpaired t‐test was performed. * p < 0.05, ** p < 0.01 and *** p < 0.001. Scale bar, C = 10 μm; E, G = 50 μm. [file ACEL-25-e70382-s002.pdf]

Figure S1

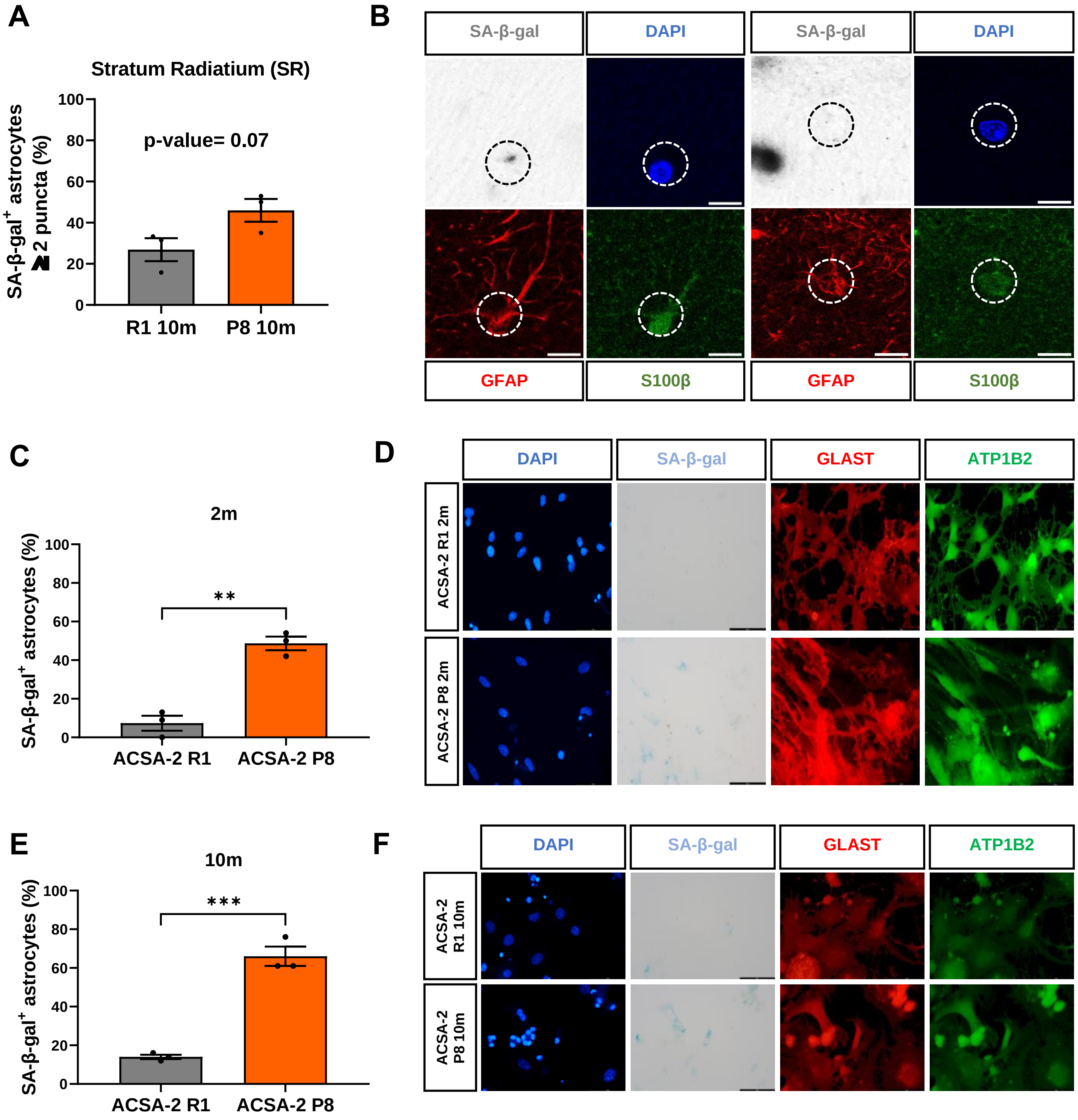
A
Stratum Radiatium (SR)
p-value= 0.07
SA-β-gal+ astrocytes ≥ 2 puncta (%)
R1 10m
P8 10m
B
SA-β-gal
DAPI
GFAP
S100β
C
2m
**
SA-β-gal+ astrocytes (%)
ACSA-2 R1
ACSA-2 P8
D
DAPI
SA-β-gal
GLAST
ATP1B2
ACSA-2 R1 2m
ACSA-2 P8 2m
E
10m
***
SA-β-gal+ astrocytes (%)
ACSA-2 R1
ACSA-2 P8
F
DAPI
SA-β-gal
GLAST
ATP1B2
ACSA-2 R1 10m
ACSA-2 P8 10m

Supplementary Figure 1. SAMP8 hippocampi are enriched in SA-β-gal$^+$ astrocytes. (A) Quantification of the percentage of astrocytes (GFAP$^+$/S100β$^+$) with two or more SA-β-gal puncta in the stratum radiatum of SAMR1 and SAMP8 mice at 10 months. (B) Representative SA-β-gal positive and negative astrocytes from stratum radiatum with GFAP (red) and S100β (green) biomarkers. The slices have 40 µm of thickness. (C) Percentage of SA-β-gal positive astrocytes (GLAST$^+$/ATP1B2$^+$) in ACSA-2 primary cultures of 2 months-old SAMR1 and SAMP8 mice. (D) Immunostaining of SA-β-gal (grey), GLAST (red) and ATP1B2 (green), in hippocampal astrocytes (ACSA-2$^+$) of 2 months-old SAMR1 and SAMP8 mice. (E) Percentage of SA-β-gal positive astrocytes (GLAST$^+$/ATP1B2$^+$) in ACSA-2 primary cultures of 10 months-old mice. (F) Immunostaining of SA-β-gal (grey), GLAST (red) and ATP1B2 (green), in hippocampal astrocytes (ACSA-2$^+$) of 10 months-old SAMR1 and SAMP8 mice. Three independent animals and primary cultures of each strain and age were analyzed (n=3). Data are presented as mean ± SEM. Unpaired t-test was performed. * $p < 0.05$, ** $p < 0.01$ and *** $p < 0.001$. Scale bar, C = 10 µm; E, G = 50 µm.
