# Supplementary material for: Astrocyte Senescence Impairs Synaptogenesis due to Thrombospondin‐1 Loss
Source: Aging Cell. 2026 Jan 18;25(2):e70382. doi: 10.1111/acel.70382 (PMC12813271; doi:10.1111/acel.70382)
Supplement: Supplementary file 2 — Figure S2: Characterization and functionality of differentiated astrocytes (Diff‐Ast) derived from neural stem cells. (A) Representative images of SAMR1 and SAMP8 Diff‐Ast using the astroglial markers GFAP (green)—S100β (red) and GLAST (green)—ATP1B2 (red). (B) Quantification of the percentage of GFAP+/S100β + double positive cells in Diff‐Ast. (C) Quantification of the percentage of GLAST+/ATP1B2+ double positive cells in Diff‐Ast. (D) Representative images of SAMR1 and SAMP8 NSCs and Diff‐Ast using the NSC marker Nestin (green). (E) Quantification of glutamate in proliferating NSCs at 2 DIV and Diff‐Ast SAMR1 and SAMP8 at 8 DIV. Data represented free glutamate in the culture medium, normalized to cell viability. (F) RT‐qPCR of Cdkn1a (p = 0.017), Il1β (p = 0.214) and Il6 (p = 0.217) in Diff‐Ast SAMR1 and SAMP8 at 11 DIV. At least three independent experiments per cell type were analyzed (n ≥ 3). Data are presented as mean ± SEM. One‐sample t‐test was performed in (D). One‐way ANOVA Tukey's multiple comparisons test was done in (E). * p < 0.05 and ** p < 0.01. Scale bar: 50 μm. [file ACEL-25-e70382-s003.pdf]

Figure S2

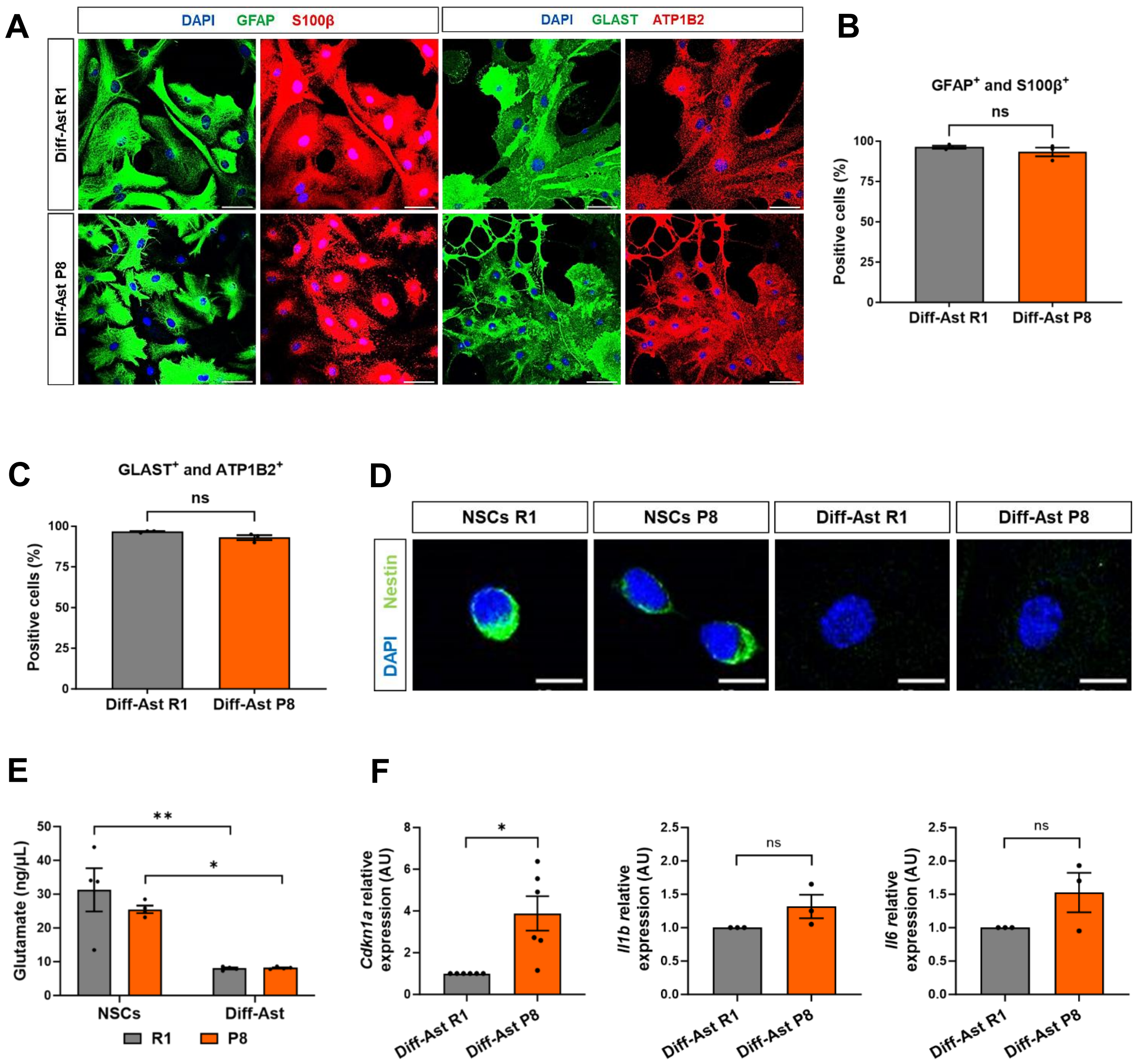
A
DAPI GFAP S100β
DAPI GLAST ATP1B2
Diff-Ast R1
Diff-Ast P8
B
GFAP+ and S100β+
ns
Positive cells (%)
Diff-Ast R1
Diff-Ast P8
C
GLAST+ and ATP1B2+
ns
Positive cells (%)
Diff-Ast R1
Diff-Ast P8
D
NSCs R1
NSCs P8
Diff-Ast R1
Diff-Ast P8
Nestin
DAPI
E
Glutamate (ng/μL)
**
*
NSCs
Diff-Ast
R1
P8
F
Cdkn1a relative expression (AU)
*
Il1b relative expression (AU)
ns
Il6 relative expression (AU)
ns
Diff-Ast R1
Diff-Ast P8

Supplementary Figure 2. Characterization and functionality of differentiated astrocytes (Diff-Ast) derived from neural stem cells. (A) Representative images of SAMR1 and SAMP8 Diff-Ast using the astroglial markers GFAP (green) - S100β (red) and GLAST (green) - ATP1B2 (red). (B) Quantification of the percentage of $GFAP^{+}/S100\beta^{+}$ double positive cells in Diff-Ast. (C) Quantification of the percentage of $GLAST^{+}/ATP1B2^{+}$ double positive cells in Diff-Ast. (D) Representative images of SAMR1 and SAMP8 NSCs and Diff-Ast using the NSC marker Nestin (green). (E) Quantification of glutamate in proliferating NSCs at 2 DIV and Diff-Ast SAMR1 and SAMP8 at 8 DIV. Data represented free glutamate in the culture medium, normalized to cell viability. (F) RT-qPCR of *Cdkn1a* ($p = 0.017$), *Il1β* ($p = 0.214$) and *Il6* ($p = 0.217$) in Diff-Ast SAMR1 and SAMP8 at 11 DIV. At least three independent experiments per cell type were analyzed ($n \leq 3$). Data are presented as mean ± SEM. One-sample t-test was performed in (D). One-way ANOVA Tukey's multiple comparisons test was done in (E). * $p < 0.05$ and ** $p < 0.01$. Scale bar: 50 µm.
