# Supplementary material for: Astrocyte Senescence Impairs Synaptogenesis due to Thrombospondin‐1 Loss
Source: Aging Cell. 2026 Jan 18;25(2):e70382. doi: 10.1111/acel.70382 (PMC12813271; doi:10.1111/acel.70382)
Supplement: Supplementary file 3 — Figure S3: Neuron survival, pre‐, and postsynaptic puncta are not affected upon ACM treatment, and TSP1/2 protein levels are decreased in SAMP8 differentiated astrocytes. (A, B) MAP2+ neuron counting in hippocampal cultures (8 random fields per culture) treated with ACM from SAMR1 and SAMP8 Diff‐Astrocytes (A) and ACM from SAMR1 and SAMP8 ACSA2+ primary astrocytes (B). (C, D) Quantification of pre‐ (VGLUT1) and postsynaptic (PSD95) vesicles in hippocampal neuron cultures treated with ACM from SAMR1 and SAMP8 Diff‐Astrocytes. (E, F) Western blot showing TSP1/2 protein levels in three independent protein lysates from SAMR1 and SAMP8 Diff‐Astrocytes, and their respective quantification. Three independent experiments were analyzed. Data are presented as mean ± SEM, and normalized to their respective controls in (C, D, and F). One‐way ANOVA Tukey's multiple comparisons test was performed in (A–D). Paired t‐test was performed in (F). * p < 0.05. [file ACEL-25-e70382-s005.pdf]

Figure S3

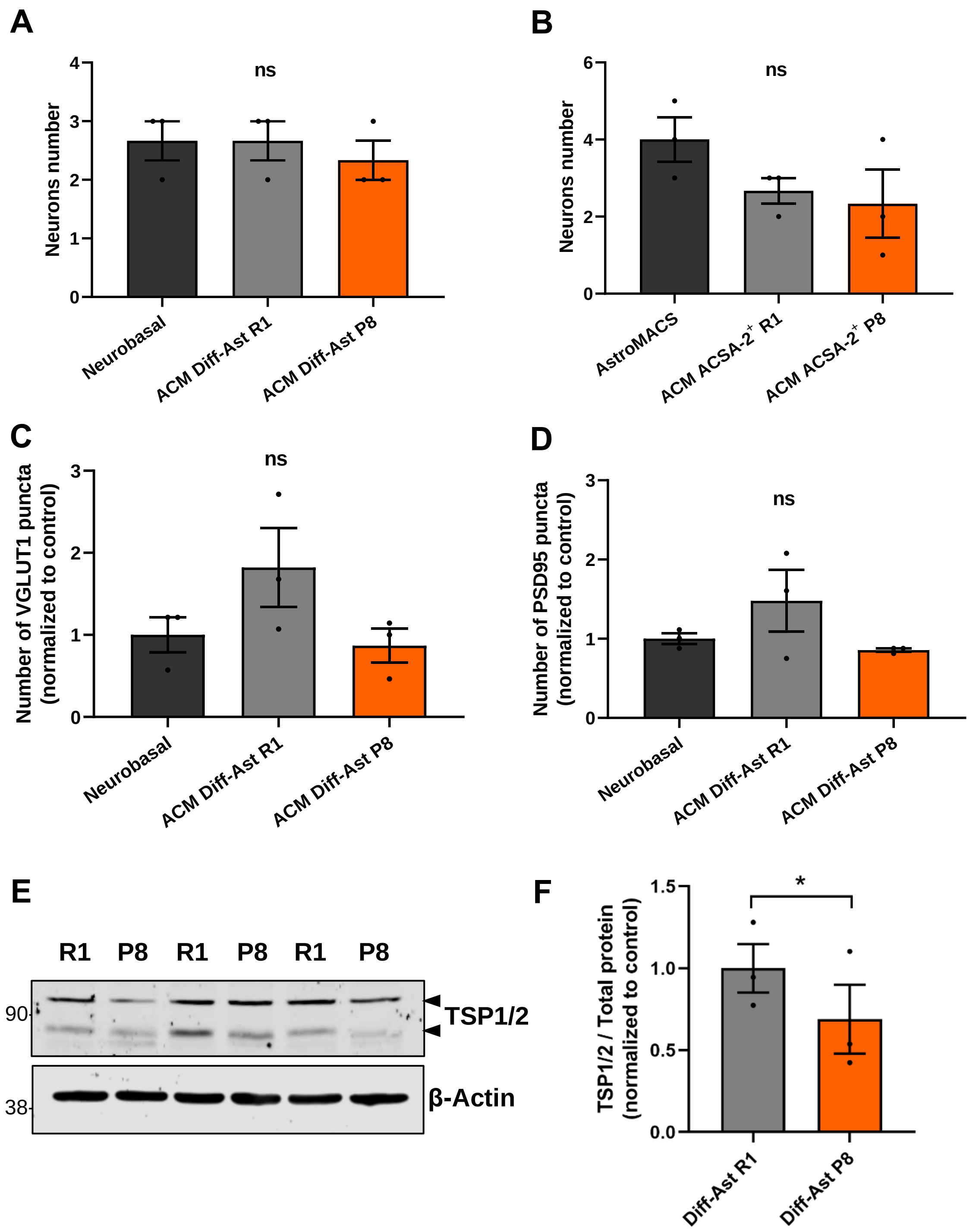

A
ns
Neurons number
0
1
2
3
4
Neurobasal
ACM Diff-Ast R1
ACM Diff-Ast P8
B
ns
Neurons number
0
2
4
6
AstroMACS
ACM ACSA-2+ R1
ACM ACSA-2+ P8
C
ns
Number of VGLUT1 puncta (normalized to control)
0
1
2
3
Neurobasal
ACM Diff-Ast R1
ACM Diff-Ast P8
D
ns
Number of PSD95 puncta (normalized to control)
0
1
2
3
Neurobasal
ACM Diff-Ast R1
ACM Diff-Ast P8
E
R1
P8
R1
P8
R1
P8
90
TSP1/2
38
β-Actin
F
*
TSP1/2 / Total protein (normalized to control)
0.0
0.5
1.0
1.5
Diff-Ast R1
Diff-Ast P8

Supplementary Figure 3. Neuron survival, pre- and postsynaptic puncta are not affected upon ACM treatment, and TSP1/2 protein levels are decreased in SAMP8 differentiated astrocytes. (A, B) $MAP2^+$ neuron counting in hippocampal cultures (8 random fields per culture) treated with ACM from SAMR1 and SAMP8 Diff-Astrocytes (A) and ACM from SAMR1 and SAMP8 $ACSA2^+$ primary astrocytes (B). (C, D) Quantification of pre- (VGLUT1) and postsynaptic (PSD95) vesicles in hippocampal neuron cultures treated with ACM from SAMR1 and SAMP8 Diff-Astrocytes. (E, F) Western blot showing TSP1/2 protein levels in three independent protein lysates from SAMR1 and SAMP8 Diff-Astrocytes, and their respective quantification. Three independent experiments were analyzed. Data are presented as mean ± SEM, and normalized to their respective controls in (C, D and F). One-way ANOVA Tukey's multiple comparisons test was performed in (A-D). Paired t-test was performed in (F). * $p < 0.05$.
