# Supplementary material for: Astrocyte Senescence Impairs Synaptogenesis due to Thrombospondin‐1 Loss
Source: Aging Cell. 2026 Jan 18;25(2):e70382. doi: 10.1111/acel.70382 (PMC12813271; doi:10.1111/acel.70382)
Supplement: Supplementary file 4 — Figure S4: Colocalization analysis shows a decrease in synapses in SAMP8 10‐month‐old hippocampal slices. (A, B) Immunostaining and quantification of excitatory pre‐ (VGLUT1, red) and postsynaptic (PSD95, green) vesicles colocalization in hippocampal tissue of SAMR1 and SAMP8 at 10‐m. Three independent animals of each strain were analyzed (n = 3). (C) Correlation analysis of VGLUT1/PSD95 co‐localized puncta, reflecting excitatory synapses, and TSP1 signal intensity normalized to the area (μm2) in Stratum Radiatum, Stratum Lacunosum Moleculare and Molecular Layer. Three independent animals of each strain were analyzed (n = 3). Spearman r = 0.7503, Two‐tailed p < 0.0001. Data are presented as mean ± SEM and normalized to SAMR1 mice. Unpaired t‐test was performed. * p < 0.05, ** p < 0.01. [file ACEL-25-e70382-s001.pdf]

Figure S4

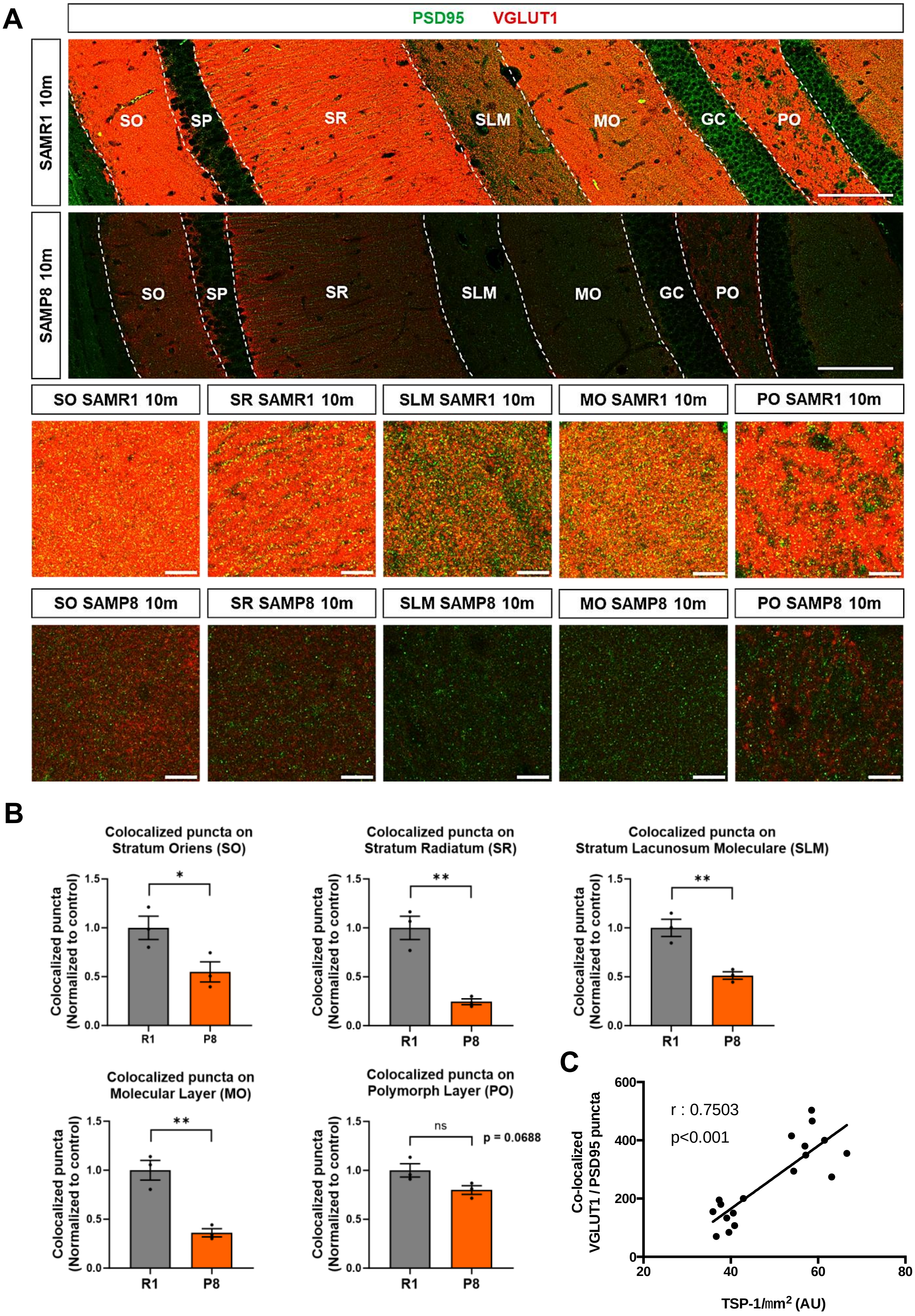

A
PSD95 VGLUT1
SAMR1 10m
SO SP SR SLM MO GC PO
SAMP8 10m
SO SP SR SLM MO GC PO
SO SAMR1 10m
SR SAMR1 10m
SLM SAMR1 10m
MO SAMR1 10m
PO SAMR1 10m
SO SAMP8 10m
SR SAMP8 10m
SLM SAMP8 10m
MO SAMP8 10m
PO SAMP8 10m
B
Colocalized puncta on Stratum Oriens (SO)
Colocalized puncta on Stratum Radiatum (SR)
Colocalized puncta on Stratum Lacunosum Moleculare (SLM)
Colocalized puncta on Molecular Layer (MO)
Colocalized puncta on Polymorph Layer (PO)
Colocalized puncta (Normalized to control)
R1
P8
ns
p = 0.0688
C
r : 0.7503
p<0.001
Co-localized VGLUT1 / PSD95 puncta
TSP-1/㎟ (AU)

Supplementary Figure 4. Colocalization analysis shows a decrease in synapses in SAMP8 10-month-old hippocampal slices. (A-B) Immunostaining and quantification of excitatory pre- (VGLUT1, red) and postsynaptic (PSD95, green) vesicles colocalization in hippocampal tissue of SAMR1 and SAMP8 at 10-m. Three independent animals of each strain were analyzed (n=3). (C) Correlation analysis of VGLUT1/PSD95 co-localized puncta, reflecting excitatory synapses, and TSP1 signal intensity normalized to the area (μm^2) in Stratum Radiatum, Stratum Lacunosum Moleculare and Molecular Layer. Three independent animals of each strain were analyzed (n=3). Spearman r = 0.7503 , Two-tailed $p < 0.0001$. Data are presented as mean ± SEM and normalized to SAMR1 mice. Unpaired t-test was performed. * $p < 0.05$, ** $p < 0.01$.
