# Supplementary material for: Astrocyte Senescence Impairs Synaptogenesis due to Thrombospondin‐1 Loss
Source: Aging Cell. 2026 Jan 18;25(2):e70382. doi: 10.1111/acel.70382 (PMC12813271; doi:10.1111/acel.70382)
Supplement: Supplementary file 5 — Figure S5: The antagonistic competitor GBP blocks TSP‐1 synaptogenic function in neurons. (A, B) Immunostaining of excitatory pre‐ (VGlut1, red) and postsynaptic (PSD95, green) vesicles colocalization in hippocampal neurons of mice primary cultures. (C) RT‐qPCR of Cacna2d1 in neuronal and non‐neuronal cells, normalized to whole hippocampus. (D) RT‐qPCR of Thbs1 in SAMP8 astrocytes, 3 days after transfection with pcDNA3.1 empty vector and pMaxGFP, or pcDNA3.1 mTSP1 and pMaxGFP (n = 2). Three independent experiments per cell type were analyzed (n = 3) in (B). Data are presented as mean ± SEM and normalized to the Neurobasal control medium in (B). One‐way ANOVA Tukey's multiple comparisons test was performed in (B). * p < 0.05. Scale bar: 50 μm. [file ACEL-25-e70382-s004.pdf]

Figure S5

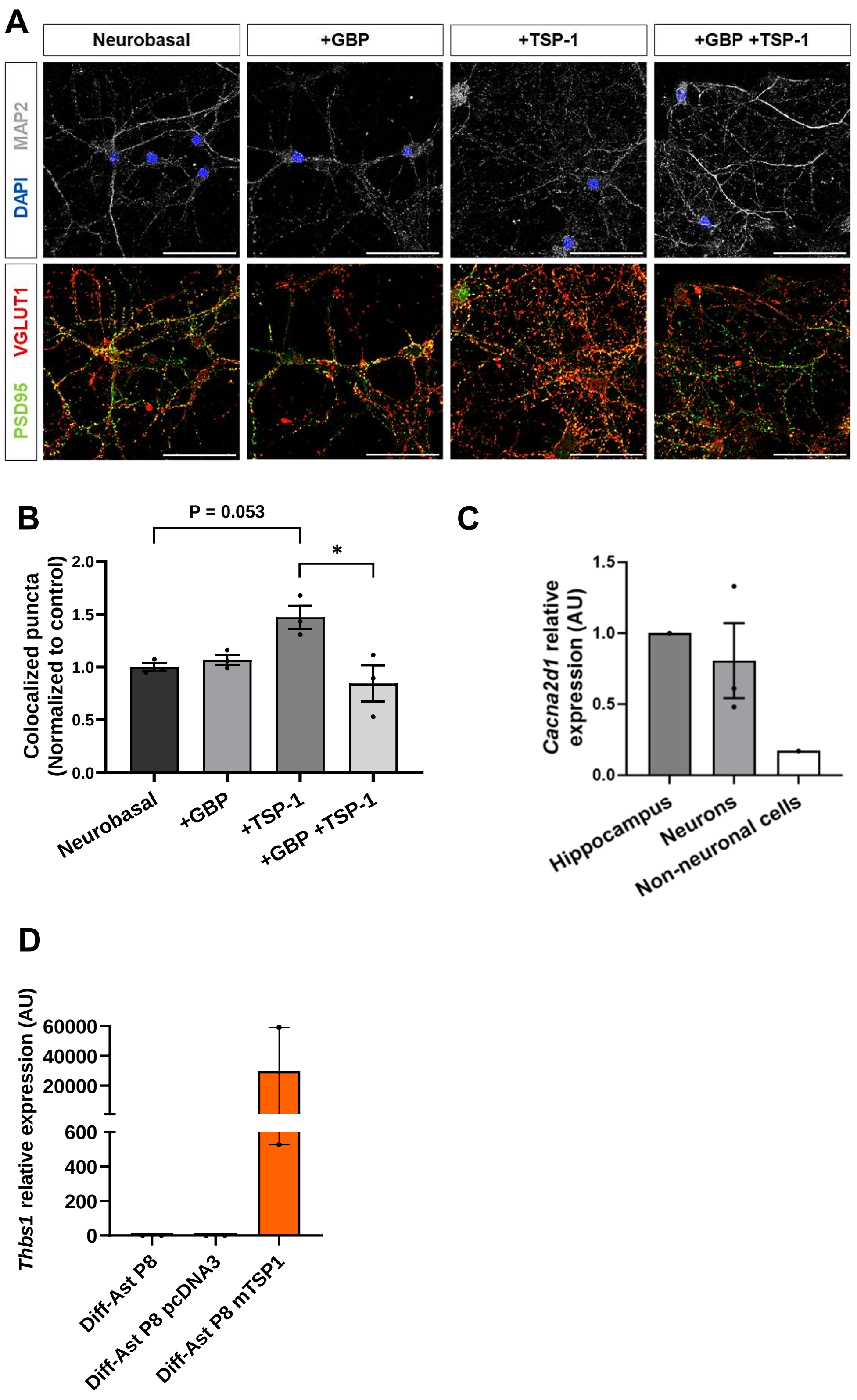
A
Neurobasal
+GBP
+TSP-1
+GBP +TSP-1
MAP2
DAPI
VGLUT1
PSD95
B
P = 0.053
*
Colocalized puncta (Normalized to control)
2.0
1.5
1.0
0.5
0.0
Neurobasal
+GBP
+TSP-1
+GBP +TSP-1
C
Cacna2d1 relative expression (AU)
1.5
1.0
0.5
0.0
Hippocampus
Neurons
Non-neuronal cells
D
Thbs1 relative expression (AU)
60000
40000
20000
600
400
200
0
Diff-Ast P8
Diff-Ast P8 pcDNA3
Diff-Ast P8 mTSP1

Supplementary Figure 5. The antagonistic competitor GBP shows a negative synaptogenic effect and TSP-1 rescues synaptic function in neurons. (A-B) Immunostaining of excitatory pre- (VGlut1, red) and postsynaptic (PSD95, green) vesicles colocalization in hippocampal neurons of mice primary cultures. (C) RT-qPCR of *Cacna2d1* in neuronal and non-neuronal cells, normalized to whole hippocampus. (D) RT-qPCR of *Thbs1* in SAMP8 astrocytes, 3 days after transfection with pcDNA3.1 empty vector and pMaxGFP, or pcDNA3.1 mTSP1 and pMaxGFP (n=2). Three independent experiments per cell type were analyzed (n=3) in (B). Data are presented as mean ± SEM and normalized to the Neurobasal control medium in (B). One-way ANOVA Tukey's multiple comparisons test was performed in (B). * $p < 0.05$. Scale bar: 50 µm.
